# Exploring the mental health effects of Universal Credit: a journey of co-production

**DOI:** 10.1177/17579139221103178

**Published:** 2022-07-14

**Authors:** M Cheetham, PJ Atkinson, M Gibson, SV Katikireddi, S Moffatt, S Morris, L Munford, F Shenton, S Wickham, P Craig

**Affiliations:** Research Fellow, National Institute for Health and Care Research (NIHR), Applied Research Collaboration North East and North Cumbria (NIHR200173), based at Department of Nursing, Midwifery and Health, Northumbria University, Coach Lane Campus East, Room H213, Newcastle-u-Tyne, NE7 7XA, UK; Public contributor, UK Poverty Truth Network; Investigator Scientist, MRC/CSO Social and Public Health Sciences Unit, University of Glasgow, Glasgow, UK; Professor of Public Health & Health Inequalities, MRC/CSO Social and Public Health Sciences Unit, University of Glasgow, Glasgow, UK; Professor of Social Gerontology, Population Health Sciences Institute, Faculty of Medical Sciences, Newcastle University, Newcastle-upon-Tyne, UK; Post Doctoral Research Associate, Population Health Sciences Institute, Faculty of Medical Sciences, Newcastle University, Newcastle-upon-Tyne, UK; Senior Lecturer in Health Economics, Division of Population Health, Health Services Research & Primary Care, School of Social Sciences, University of Manchester, Manchester, UK; Public Involvement and Community Engagement Manager, National Institute for Health and Care Research (NIHR), Applied Research Collaboration North East and North Cumbria (NIHR200173), based at CNTW NHS Foundation Trust, St Nicholas’ Hospital, Newcastle Upon Tyne, NE3 3XT, UK; Wellcome Trust Research Fellow, Department of Public Health, Policy & Systems, Institute of Population Health, University of Liverpool, Liverpool, UK; Professor of Public Health Evaluation, Inequalities and Health, MRC/CSO Social and Public Health Sciences Unit, University of Glasgow, Glasgow, UK

This article offers reflections and experiences of public engagement in a National Institute for Health Research funded study about the mental health effects of Universal Credit.

PJ’s poem powerfully illustrates his experiences of Universal Credit (UC). In this article, we outline our approach to public involvement and engagement (PIE) in a mixed-method, multi-site study about the mental health effects of UC funded by the National Institute for Health Research (NIHR).

Public involvement in research is defined by NIHR as ‘an active partnership between members of the public and researchers in the research process’. We view public engagement as a social practice of dialogue and learning between researchers and the public;^1^ at its heart is the core value of social justice, shaped by wider societal developments towards realising citizen empowerment.^2^ We adopted the term PIE in preference to the more commonly used patient and public involvement, given that our study involves citizens/people with experience of UC and staff supporting them. Deciding who our relevant ‘publics’ are, and how we meaningfully involve them in the research is evolving over time. Here, we describe and reflect on the ongoing process of PIE in the context of this four-year research project.

## Background and Introduction

This study began in May 2021, but the public involvement process started long before in 2016 in North East England when the public, voluntary sector staff and elected members in local government began voicing concerns about the rollout of UC and its consequences for citizens and services. This coincided with MC working as an embedded researcher in Gateshead Council Public Health team who, in response to these concerns, commissioned qualitative research that subsequently reported negative experiences of UC.^3^ Inspired by powerful narratives of people claiming UC, including PJ, MC developed links with Gateshead Poverty Truth Commission (GPTC). Their approach centred on building connections between people with lived experience and those in positions of power to affect change. Collaboration between academics with a strong track record of previous work highlighting the health impacts of UK welfare reforms over the last decade,^4–9^ enabled a successful application to NIHR’s call for research on changes to the welfare system (19/106). Long-standing partnerships between the research team, citizens and staff in voluntary organisations and local government informed the application.

**Box 1.** A poem by PJ.

**The Road** Why does my benefit … CRUSH down. The road to employed is a steep enough hill, why place a mountain to defeat my will. Why does my benefit … CRUSH down. The road to good health, is long and hard to chart, why place a minefield to blow me apart. Why does my benefit . . . CRUSH down. The road to inclusion is digital only, why place obstacles to hinder and goad me. Why does my benefit. . .CRUSH down The road out of poverty is a torrid time, why do I feel I did a crime. Why does my benefit … CRUSH down. The road they built doesn’t care or feel, I’m not a problem I’m just real. Why does my benefit . . . CRUSH ME DOWN.

## Who is involved?

Research team members drew on existing links with stakeholders and UC claimants in North East England, Liverpool and Glasgow whose knowledge and lived experience were valued equally. We anticipated input would benefit the research in multiple ways: help prioritise the questions we ask in the research, identify outcomes of interest, and enhance the quality and relevance of the findings. Although we took a rights-based approach, and were aware of NIHR’s emphasis on paid involvement as a research funder,^10^ we were (and remain) concerned about the practical and personal risks for UC claimants, including on entitlement, eligibility and conditionality. These risks, which we have discussed with colleagues in the Department for Work and Pensions (DWP), are outlined in Box 2.

**Box 2.** Risks of public involvement and engagement for Universal Credit claimants.

Universal Credit claimants may already be navigating complex Department for Work and Pensions (DWP) rules about payments and conditionality. A principle of conditionality holds that that access to publicly funded welfare benefits, like Universal Credit, should be dependent on an individual agreeing to meet particular obligations.^11^ Universal Credit claimants are required to undertake set amounts of work search activities each week. Claimants can face sanctions (where their benefit is stopped temporarily) for perceived breaches of the claimant commitment negotiated with their work coach. Public involvement and engagement activity could affect actual or perceived availability for work. Our previous research showed variability in enforcement/interpretation of Universal Credit rules, resulting in unpredictable decision-making with serious potential consequences for claimants. Tensions exist between Universal Credit rules and NIHR requirements to pay public contributors set amounts for public involvement activities. We found it is important to distinguish between vouchers given for participation in research interviews versus reimbursement of expenses versus remuneration for public involvement and engagement activities. Payments for public involvement activities could count as earned income and could affect Universal Credit entitlement. We advised claimants to seek independent advice about their specific circumstances from welfare rights services. The perception of claimants’ involvement in ‘paid work’ (public involvement and engagement activities) could threaten their Universal Credit entitlements more broadly, or claimants previously assessed as having ‘limited capability for work’ could be seen as ‘fit for work’ following engagement in public involvement activity.

## Our Approach to Pie

We set out our approach to PIE in a jointly agreed values statement (see Supplementary Material 1). We used the Public Involvement Impact Assessment Framework^12^ to stimulate discussions about the aims and intended impact of public involvement in each work package. Recognising the need for flexibility, we are working with UC claimants and stakeholders to explore how they want to be involved and to date have captured these in a menu of options (see practical activities in Supplementary Material 1). We discussed these with the Department for Work and Pensions (DWP) and support organisations in efforts to reduce the potential risks of PIE activities. We obtained letters explaining public involvement that UC claimants can use if questioned by Job Centre staff or work coaches. Our budget included payment for public involvement activities according to the NIHR guidance. A set of payment options was offered to minimise the risks for UC claimants who chose to be involved. Guided by advice from Citizens Advice and DWP, we included options to receive expenses only, or payments to be made to voluntary and community sector (VCS) organisations (a copy of our PIE payment policy is available in Supplementary Material 2).

Early on, we consulted UC claimants, advice workers, public involvement leads and Universal Credit Essentials (UCE; an education and advice charity started and run by current and former UC claimants). UCE had input during the proposal development process, including commenting on the overall research plan as described in the plain language summary. We simplified qualitative fieldwork documents following advice from public partners and welfare rights colleagues and augmented the written materials with a short film, co-produced with public engagement partners.

Our public contributors encouraged us to revise the Privacy Notice, to improve accessibility generally and specifically to ensure clarity on the nature of harms that may require confidentiality to be breached and what action would be taken in that event. The process of ratifying the new version with University colleagues responsible for data protection and ethics seems to have highlighted the value of public engagement and may lead to some changes at an institutional level to ensure the accessibility of public documents. Our discussions with colleagues in finance as a result of public involvement have resulted in changes to claims forms to ensure they are fit for purpose.

Our public involvement activities included an opportunity to be involved in the recruitment and selection panel for a new researcher working on the study. Following his involvement, PJ offers his thoughts on co-production in Box 3.

**Box 3.** PJ’s thoughts on co-production.

After a second relapse of my mental health in 2019, one of the main attributes of my improvement had been my joining the Poverty Truth Commission in Gateshead, as a community or life experience commissioner, relaying my story of my interactions with Universal Credit. This had culminated in a high point when we had our launch event in March 2020. The offer to take part in co-production of the Universal Credit research project was therapeutic, but also made me feel useful. I have not worked for seven years, so the keeping of diaries, attending meetings, helping shape the questionnaire and being on the recruitment panel for the North East researcher made me feel my lived experience felt both important and valued, and I felt better in myself. This has led me to feel very strongly on the value of co-production, and the effort it requires to do it properly. Taking information from people who are vulnerable, lack confidence, are suffering mental health, addiction or are of poor education requires patience and empathy, but the information received is ‘pure gold’. Only a person living in their situation can give the insight that they bring. Being part of the recruitment panel allowed me to offer a non-professional, or technical view; was the person warm and nice to speak to, would I want to tell them my story? Did they listen well and understand how they were going to approach this qualitative research and were they open to co-producer’s input?

## How our Approach is Evolving

We are at the beginning rather than the end of the journey and anticipate public involvement activities will continue to develop throughout the study, across all workstreams. PIE is a standing item at monthly team meetings, and all researchers are encouraged to keep an impact log. One of our aims is to open up the possibilities of PIE, and we continue to reflect on our efforts. We are adapting our approach to PIE to take account of people’s needs and concerns about digital exclusion during COVID. Sometimes this means taking a walk in the park instead of organising an online meeting.

The research team are listening, learning and creating opportunities for others to hear about the effects of UC through poetry, conversation and continual dialogue. We are hoping to change assumptions, narratives and perspectives along the way. We remain alert to differences between stated policy and on-the-ground implementation, particularly following conversations with UCE that Scottish Choices Universal Credit payment arrangements and Alternative Payment Arrangements in the rest of the UK are not markedly different and are often dictated by work coaches at local level.

Our PIE payment processes have been developed in conjunction with public contributors, to establish their preferred methods of payment using guidance about how different kinds of payment will be assessed and taken into account by DWP/Job Centre staff. Colleagues operating university payment and claims systems are open to adapting systems so that they fit the specific requirements of our study public partners. The research team are committed to sharing our learning throughout the study, including developing a publication policy to reflect our learning of co-authoring papers in collaboration with public contributors.

## Reflections and Recommendations for Researchers and Research Funders

We are aware that there are limits to the changes that can occur as a result of PIE (e.g. study design approved by funders and requirements for inclusion of material on information leaflets). We aim to be transparent about the limits of influence in the study. Members of the research team built on our previous relationships with practitioners, policy-makers and people with experience of UC. The study benefitted from this early engagement. However, challenges remain in offering meaningful PIE opportunities *before* formal research funding begins. Pump priming funding for researchers to have capacity to start these processes before an application/award begins would be beneficial. Time is needed for meaningful co-production to be factored into research designs. Our experience has demonstrated the immense contributions of voluntary and community organisations that provide support for people involved in research.

Working together on a Public Involvement and Engagement Values Framework helped build trust and shared understanding between team members, stakeholders and public contributors. Anticipating potential risks of harm added layers of complexity.^13^ Paying close attention early on to remuneration issues helped reduce potential adverse impact on UC claimants. We remain concerned that, depending on UC claimants’ circumstances, NIHR recommended payment rates could cause significant harm to some of the people we most want to engage in research. For this reason, we remain vigilant about the potential costs to public partners,^14^ and seek ways to reduce the possibility of involvement exacerbating/widening existing health inequalities.

Public involvement enhanced the researcher recruitment and selection process in this study and should become routine practice in university appointments for publicly funded research. We acknowledge the structural difficulties of sharing power in the context of the existing research hierarchy within which co-production commonly takes place.^15^ Our experience has shown that PIE can disrupt taken for granted assumptions, values and norms if people are open to change and differing perspectives. Capturing these outside our multi-disciplinary research team is not straightforward.^16^

## Conclusion

Undertaking research on UC requires a focus on the perspectives of communities most at-risk.^2^ None of the research team members consider themselves experts in public involvement or co-production. Our approach has been characterised by humility and a willingness to try new approaches, build new relationships, listen and learn from experience. PIE is firmly established in our ongoing research, which enables regular reflection as well as acknowledging and addressing the possibilities of unintended consequences. We anticipate more bumps in the road. While hopefully we may have contributed by outlining our approach, we are aware that the existing ‘hierarchies of academic knowledge production’^17^ make it challenging to fully realise the transformative potential of publicly engaged research.

## Supplemental Material

sj-docx-1-rsh-10.1177_17579139221103178 – Supplemental material for Exploring the mental health effects of Universal Credit: a journey of co-production Click here for additional data file. Supplemental material, sj-docx-1-rsh-10.1177_17579139221103178 for Exploring the mental health effects of Universal Credit: a journey of co-production by M Cheetham, PJ Atkinson, M Gibson, SV Katikireddi, S Moffatt, S Morris, L Munford, F Shenton, S Wickham and P Craig in Perspectives in Public Health

sj-docx-2-rsh-10.1177_17579139221103178 – Supplemental material for Exploring the mental health effects of Universal Credit: a journey of co-production Click here for additional data file. Supplemental material, sj-docx-2-rsh-10.1177_17579139221103178 for Exploring the mental health effects of Universal Credit: a journey of co-production by M Cheetham, PJ Atkinson, M Gibson, SV Katikireddi, S Moffatt, S Morris, L Munford, F Shenton, S Wickham and P Craig in Perspectives in Public Health
