# Supplementary material for: Exploring the mental health effects of Universal Credit: a journey of co-production
Source: Perspect Public Health. 2022 Jul 14;142(4):209–12. doi: 10.1177/17579139221103178 (PMC9284081; doi:10.1177/17579139221103178)
Supplement: sj-docx-1-rsh-10.1177_17579139221103178 – Supplemental material for Exploring the mental health effects of Universal Credit: a journey of co-production [file sj-docx-1-rsh-10.1177_17579139221103178.docx]

**Supplementary material 2**

**Evaluation of the health impacts of Universal Credit (NIHR131709)**

**Public Involvement and Engagement (PIE) Values Framework**

The overall aims of public involvement and engagement (PIE) in this study are to ensure:

- The research addresses the primary questions of interest for the public, claimants and their advocates, policy makers and practitioners
- Public members have meaningful opportunities to guide and inform the research process
- Data analysis makes sense
- Findings are presented in ways that are accessible for target audiences.

**Who are public members for this study?**

People with experience of claiming Universal Credit (UC), policy makers and practitioners with working knowledge of UC implementation.

**Our approach to Public Involvement and Engagement**

The research team will build on and maintain working relationships with organisations and networks supporting UC claimants and other stakeholders through regular communication. Formal and informal opportunities for involvement and engagement will be offered through existing partnerships in the North East and North West of England and West of Scotland.

The research team will seek active input of those with experience of claiming UC, policy makers and practitioners with working knowledge of UC implementation to:

- Provide timely insights about how any changes to UC policy play out in practice.
- Advise on feasibility of research methods, provide a sounding board for researchers to test ideas as each work package (WP) progresses.
- Consider potential positive and negative consequences of the implementation of UC on claimants and their families.
- Contribute knowledge and understanding of the policy levers, mediating mechanisms and equity characteristics that are important in UC.
- Guide researchers to ensure inclusion of people who may be affected by the introduction of UC.
- Ensure diverse views are sought, including members of groups adversely affected by health and social inequalities.
- Provide links with wider networks.

Practical activities may include the following:

- Shape and refine the logic model for the study.
- Review the values framework developed to inform public involvement and engagement (PIE).
- Inform the development of ethics applications and supporting documents.
- Contribute to the recruitment and selection process of researchers working on the study.
- Contribute to the development of research materials, including Participant Information Sheets, consent forms, topic guides and other documentation for WP3.
- Facilitate, guide and support sampling and recruitment of research participants and suggest practical solutions to issues encountered by the team.
- Contribute to data analysis and sense checking of early findings.
- Co-author publications and jointly present at conferences and events.
- Build support and capacity among UC claimants to undertake their own independent research

Our approach is informed by the [NENC ARC (2021) strategy for public involvement and engagement](https://arc-nenc.nihr.ac.uk/wp-content/uploads/2020/09/Guide-How-we-are-developing-a-Public-Involvement-and-Community-Engagement-Strategy-for-our-ARC.pdf), and evidence of what works in developing and maintaining effective public involvement. The research team will support members of the public to contribute to the research through a range of activities to optimise the relevance, implementation and dissemination of research. Regular opportunities for PIE will be provided online and, where possible, through PIE meetings in each study locality. The research team see these interactions as vital to the effective conduct of the study.

Subject to COVID restrictions, the format and frequency of face-to-face meetings will be negotiated with public members, who will be reimbursed for their contribution according to [NIHR guidance (2021),](https://www.nihr.ac.uk/documents/payment-guidance-for-members-of-the-public-considering-involvement-in-research/27372) avoiding any potentially adverse effects on UC entitlement or eligibility.

We will provide UC claimants with a letter for DWP / their Work Coach as recommended in the NIHR guidance (2021). We recognise that a donation directly to a group or support organisation may be preferable, offering control over how the money is used without affecting individual’s benefit entitlement or eligibility. In the event of any difficulties/adverse impact on any individual case, this will be taken up with the NIHR Benefits helpline if necessary.

The research team have co-developed the values framework underpinning their approach to PIE, using the Public Involvement Impact Assessment Framework (Piiaf) (Popay and Collins 2014) <https://piiaf.org.uk/documents/values-summary.pdf>. This has been discussed and agreed by all research team members.

By the start of fieldwork, we will have worked through the questions in the Public Involvement Impact Assessment Framework (<http://piiaf.org.uk/documents/exec-summary-0114.pdf>) agreed the PIE methods the team will use, identified potential barriers and facilitators, and negotiated practical issues re. funding, remuneration and reimbursement with University finance departments. A payment policy has been drafted and agreed by stakeholders.

We will agree a consistent approach to recording our PIE activities, including any unintended consequences, piloting the use of PIE Impact logs and the GRIPP2 short form available here <https://www.bmj.com/content/358/bmj.j3453> . This will be reviewed after 3 months.

**Values Framework**

The research team believe:

- Effective PIE is of intrinsic value. It is a fundamental human right to have a say, and for public views to be heard and taken into account in the conduct of research.
- UC claimants and staff supporting them have a right to be involved in meaningful opportunities to shape the research to ensure its relevance

The research team will:

- Allocate sufficient time for meaningful public involvement throughout the research process
- Communicate clearly from the outset with members of the public
- Respect public contributors’ rights to confidentiality
- Take account of the views of public members in the conduct of the research
- Respect the diversity of values, skills, knowledge and experience people bring
- Value, acknowledge and reward public involvement.
- Negotiate reimbursement for expenses and costs of taking part, to ensure individuals are not out of pocket, in collaboration with UC claimants and staff supporting them, respecting individual wishes and circumstances
- Seek ways to involve UC claimants and staff supporting them in ways which minimise the risks of harm for those who chose to be involved.
- Explain potential risks of PIE including financial harm, eligibility or entitlement to UC, health and wellbeing of public contributors
- Aim to resolve disagreements or differences of opinion in open, honest, transparent ways
- Host research activities, and provide opportunities to contribute to joint analysis, interpretation of findings and development of recommendations to ensure they make sense
- Seek public involvement in dissemination strategies to ensure they are accessible for diverse target audiences
- Ensure that involvement is not overly burdensome for PIE partners
- Enable public contributors to stop their involvement at any point without giving reasons
- Communicate and keep records of changes made in response to PIE activities
- Seek feedback about the experience of PIE in this study, including any unintended or adverse consequences, and take steps to mitigate future risks
- Adhere to the NIHR policy on preventing harm in research in undertaking PIE activities <https://www.nihr.ac.uk/documents/nihr-policy-on-preventing-harm-in-research/27567>

Researchers, academics, policy and practice partners involved in the Advisory Group and Study Steering Committee will be encouraged to use the values framework to inform and review the involvement and engagement activities undertaken as part of the study.

**References**

ESRC Framework for Research Ethics (<https://esrc.ukri.org>)

Pandya-Wood et al. (2017) A framework for public involvement at the design stage of NHS health and social care research: time to develop ethically conscious standards *Research Involvement and Engagement* (2017) 3:6 DOI 10.1186/s40900-017-0058-y

Popay and Collins (2014) <https://piiaf.org.uk/documents/values-summary.pdf>.

NIHR Policy on preventing harm in research (2021) <https://www.nihr.ac.uk/documents/nihr-policy-on-preventing-harm-in-research/27567>
