# Supplementary material for: Exploring the mental health effects of Universal Credit: a journey of co-production
Source: Perspect Public Health. 2022 Jul 14;142(4):209–12. doi: 10.1177/17579139221103178 (PMC9284081; doi:10.1177/17579139221103178)
Supplement: sj-docx-2-rsh-10.1177_17579139221103178 – Supplemental material for Exploring the mental health effects of Universal Credit: a journey of co-production [file sj-docx-2-rsh-10.1177_17579139221103178.docx]

**Supplementary material 2**

**NIHR Universal Credit Study Public Involvement and Engagement (PIE) Payment Policy**

<https://fundingawards.nihr.ac.uk/award/NIHR131709>

As set out in the NIHR guidance on payment <https://www.nihr.ac.uk/documents/payment-guidance-for-researchers-and-professionals/27392>, the Universal Credit study team are committed to having a clear policy on payment and recognition in place prior to involvement, so that members of the public know in advance what is being offered and are able to make informed decisions about getting involved.

The research team aims to provide clear and consistent information to members of the public involved in the NIHR funded study on Universal Credit about what to expect before, during and after public involvement. This document includes the processes for members of the public involved in the Universal Credit study to claim payment and expenses.

This policy has been developed with the involvement and agreement of Glasgow University and Northumbria University finance, payroll and HR departments to ensure it can be implemented using existing systems.

The research team have sought advice from the Department for Work and Pensions (DWP), from members of the public who are UC claimants and from staff supporting them, including an expert welfare benefits adviser, on the conditions that must be followed by members of the public who are in receipt of state benefits, including Universal Credit.

The research team have included measures to alleviate potential benefit barriers and to prevent misunderstandings with Jobcentres that may lead to benefits being stopped or reviewed. A letter can be provided for UC claimants involved in the study to show their Work Coach.

This letter states that good practice guidance for service user involvement in research recommends that members of the public should be offered payment for activities such as attending meetings and events, and that reasonable out-of-pocket expenses should be covered. It notes that:

• as involvement activities are often arranged on an ad hoc basis, organisations often pay service users monthly in arrears

• to assist service users who receive welfare benefits which have earnings limits or disregards, organisations offer involvement on a voluntary basis, or at a lesser amount, if requested

• as the payment period may be over a month or more, we anticipate that Jobcentre Plus will treat these payments as averaged over the payment period. (See DMG Chapter 48 paras 4080-81 and for a cycle of work para 48094).

• the Department for Work and Pensions legislation exempts ‘service users and carers’ from the application of notional earnings and treats reimbursed expenses for service user involvement as ignored (see ADM Chapter H3, paras 3160 and 3241)

• service users can withdraw from involvement activities at any time to attend ‘employment-related’ activities

The PIE payment policy for the evaluation of the health impacts of Universal Credit study will adopt the payment rates suggested by NIHR in their payment [guidance](https://www.nihr.ac.uk/documents/payment-guidance-for-researchers-and-professionals/27392). The amounts offered vary according to activities undertaken. Examples of payment rates for involvement activities members of the public should expect to be offered for each activity have been categorised and the payment rates allocated to these are set out [here](https://www.nihr.ac.uk/documents/payment-guidance-for-researchers-and-professionals/27392).

The research team will offer members of the public the option of asking for payment at a lower amount or declining a payment and offering to volunteer or offering payment to a specific charity or voluntary organisation of their choice.

The Public Involvement and Engagement Values Framework developed for the study provides information about the nature of the involvement opportunities available. The research team will negotiate the nature, duration and frequency of involvement with members of the public involved and will ensure that expenses are covered so no-one is out of pocket as a result of their involvement.

The expenses which can be reimbursed or covered according to standard organisational policy include:

- Mileage allowance according to current [HMRC guidance](https://www.gov.uk/government/publications/rates-and-allowances-travel-mileage-and-fuel-allowances)
- Travel, transport and subsistence rates according to [HMRC guidance](https://www.gov.uk/hmrc-internal-manuals/employment-income-manual/eim05231)
- Travel expenses, taxi, childcare, carer, personal assistant costs may be paid for in advance where necessary.

The process of claiming payments and expenses is set out here. With agreement by relevant research team / work package lead, members of the public will be asked to complete a Northumbria University claim for involvement form (available on request from the study PIE lead Mandy Cheetham).

Options for rewarding involvement will be agreed as follows and the relevant sections of the form completed and returned to Mandy.Cheetham@Northumbria.ac.uk for processing:

- Hourly rate of pay processed via the University payroll system (using NIHR rates)
- Voluntary, no payment required.
- Payment to another nominated organisation.
- Out of pocket expenses only.

If members of the public opt for payment via payroll, we have been advised that they may be asked for evidence of right to work in the UK to be verified by the University.

The cut-off dates for organisational systems, mean that forms need to be submitted by the 5^th^ of the month to ensure payment will be received the following month.

Members of the public who receive benefits, including Universal Credit will be advised that different benefit conditions and payment limits or disregards exist depending on their particular sets of circumstances.

The research team will advise a person who is registered self-employed that they will be responsible for their own tax. UC claimants will be advised about sources of personalised advice (see below).

Welfare Rights Advice for individuals can be obtained free from local Citizens Advice services

Citizens Advice National Advice line England Tel. 0800 144 8848 Monday – Friday 9am-5pm

Citizens Advice National Advice Line Scotland Tel. 0800 028 1456 Monday – Friday 9am-5pm

or by contacting [mandy.cheetham@northumbria.ac.uk](mailto:mandy.cheetham@northumbria.ac.uk) from the research team who can request a ticket number for the Welfare Rights Service at Citizens Advice Bedford Tel. 01234 867944. A phone appointment can then be arranged individually or with support from the research team.

A tailored letter from the research team will be provided on request for UC claimants who are involved in PIE to prevent misunderstandings with Jobcentre Plus staff. The letter will:

- explain how involvement is different to employment and about support for mobility or care needs
- allow members of the public who receive state benefits sufficient time to obtain reliable advice on their benefit conditions and to consider what level of involvement and payment they can accept, and sufficient time to follow required Jobcentre Plus procedures

The PI and research team members will regularly review the PIE payment policy to:

- ensure that staff who administer the policy have relevant training and ongoing support
- the policy is updated regularly to ensure that it remains relevant
- ensure any problems are resolved promptly and efficiently

We aim to write in clear plain language, and welcome feedback if we have not achieved this, or if other formats are required.

We will adhere to the NIHR policy on preventing harm in research in undertaking PIE activities <https://www.nihr.ac.uk/documents/nihr-policy-on-preventing-harm-in-research/27567>.

Mandy Cheetham (PIE lead) or Peter Craig (Principle Investigator) can be contacted if there is a problem with payment or expenses for the study.

Contact details

[Mandy.cheetham@northumbria.ac.uk](mailto:Mandy.cheetham@northumbria.ac.uk)

[Peter.Craig@glasgow.ac.uk](mailto:Peter.Craig@glasgow.ac.uk)

Further information about DWP guidance is available here

DWP decision makers guidance [Advice for decision making: staff guide - GOV.UK (www.gov.uk)](https://eur02.safelinks.protection.outlook.com/?url=https%3A%2F%2Fwww.gov.uk%2Fgovernment%2Fpublications%2Fadvice-for-decision-making-staff-guide&data=04%7C01%7Cmandy.cheetham%40northumbria.ac.uk%7C500a0a290b364448551108d9452dae64%7Ce757cfdd1f354457af8f7c9c6b1437e3%7C0%7C0%7C637616887910622032%7CUnknown%7CTWFpbGZsb3d8eyJWIjoiMC4wLjAwMDAiLCJQIjoiV2luMzIiLCJBTiI6Ik1haWwiLCJXVCI6Mn0%3D%7C0&sdata=PPJXQqp47TQ8h3bxNozKxjYrT%2FNLSAXUmFP%2FnbuVwjE%3D&reserved=0)

Draft 2.

Review date January 2022.
